# *Schistosoma haematobium* infection status and its associated risk factors among pregnant women in Munyenge, South West Region, Cameroon following scale-up of communal piped water sources from 2014 to 2017: a cross-sectional study

**DOI:** 10.1186/s12889-019-6659-7

**Published:** 2019-04-11

**Authors:** Godlove Bunda Wepnje, Judith Kuoh Anchang-Kimbi, Vicky Daonyle Ndassi, Leopold Gustave Lehman, Helen Kuokuo Kimbi

**Affiliations:** 1 Department of Zoology and Animal Physiology, Faculty of Science, University of Buea, P.O. Box 63, Buea, Cameroon; 2 Department of Animal Biology, Faculty of Science, University of Douala, P.O. Box 24157, Douala, Cameroon; 3 Department of Medical Laboratory Science, Faculty of Health Sciences, University of Bamenda, P.O. Box 39, Bambili, Cameroon

**Keywords:** UGS, Pregnancy, Piped water source, Stream usage and contact behaviour

## Abstract

**Background:**

In 2014, a study in Munyenge revealed a high prevalence of urogenital schistosomiasis (UGS) among pregnant women. This study investigated he prevalence and risk factors of UGS in pregnancy following scale-up of piped water sources from 2014 to 2017. Secondly, we compared stream usage, stream contact behaviour, infection rate and intensity with the findings of 2014.

**Methods:**

Consenting pregnant women reporting for antenatal care (ANC) in the different health facilities were enrolled consecutively between November 2016 and January 2018. Information on age, gravidity status, residence, marital status, educational level, occupation, household water source, frequency of contact with water and stream activities were obtained using a semi-structured questionnaire. Urine samples were examined for the presence of microhaematuria and *S. haematobium* ova using test strip and filtration/microscopy methods respectively. Data were analysed using univariate and multivariate regression analyses and relative risk reductions calculated.

**Results:**

Of the 368 women enrolled, 22.3% (82) were diagnosed with UGS. Marital status (single) (aOR = 2.24, 95% CI: 1.04–4.79), primary - level of education (aOR = 2.0; 95% CI: 1.04–3.85) and domestic activity and bathing in the stream (aOR = 3.3; 95% CI: 1.83–6.01) increased risk of *S. haematobium* infection. Meanwhile, fewer visits (< 3 visits/week) to stream (aOR = 0.35, 95% CI = 0.17–0.74) reduced exposure to infection. Piped water usage was associated with reduced stream usage and eliminated the risk of infection among women who used safe water only. Compared with the findings of 2014, stream usage (RRR = 23 95% CI: 19–28), frequency (≥ 3 visits) (RRR = 69 95% CI: 59–77) and intensity of contact with water (RRR = 37 95% CI = 22–49) has reduced. Similarly, we observed a decrease in infection rate (RRR = 52, 95% CI = 40–62) and prevalence of heavy egg intensity (RRR = 71, 95% CI = 53–81).

**Conclusion:**

Following increased piped water sources in Munyenge, *S. haematobium* infection has declined due to reduced stream contact. This has important implication in the control of UGS in pregnancy.

**Electronic supplementary material:**

The online version of this article (10.1186/s12889-019-6659-7) contains supplementary material, which is available to authorized users.

## Background

Schistosomiasis is a chronic parasitic disease caused by blood flukes of the genus *Schistosoma* and transmitted by snails found in fresh water bodies that have been contaminated by *Schistosoma* eggs. People become infected during dermal contact with water containing schistosome cercariae. In endemic areas, where there is lack of adequate water supply, poverty, ignorance and poor hygienic practices, children, women, fishermen and farmers are the high risk groups for schistosomiasis [1–4]. Women, in particular, are more likely to be exposed to infection during activities carried out in streams such as domestic activities including washing clothes, fetching water and bathing [5, 6]. It is estimated that approximately 40 million women of childbearing age are infected with schistosomiasis, with almost 10 million infected pregnant women in Africa [1, 7]. Increasingly, findings from several studies suggest that schistosomiasis in pregnancy is an area of major public health concern [6, 8–13].

*Schistosoma haematobium* is prevalent in Africa and Middle East, where the infection is causing significant morbidity and mortality when compared with *S. mansoni*. Schistosome eggs deposited in the wall of the urogenital bladder [14] release highly inflammatory antigens [15], triggering granuloma formation, a range of urothelial abnormalities and related signs such as haematuria, dysuria and lesions of the bladder, kidney failure and bladder cancer [16]. Several studies have reported associations between UGS and HIV [17–19] and increasing evidence supports that it is a plausible risk factor for HIV acquisition [20]. In pregnancy, UGS has been associated with severe anaemia [1] particularly in co-infection with *P. falciparum* [6], maternal mortality [8, 21], premature birth and low birth weight [13, 22]. Drug-based control of morbidity related to infection has been the primary WHO strategy for schistosomiasis control, with treatment given mainly through community and school-based mass treatment with praziquantel [23]. Older age groups, including pregnant women are often left untreated. Despite recent evidence of the safety of praziquantel in human pregnancy, barriers to adopting polices for such treatment still remain [24]. Consequently, affected pregnant women can serve as reservoirs for infection bringing the distribution of the disease to pre-control level over time. More so, morbidity that builds in untreated pregnant women may result in poor pregnancy outcomes [25]. To achieve sustainable control (elimination or eradication of schistosomiasis), improvements of water, sanitation, hygiene infrastructure and modification of risk behaviour are necessary to prevent transmission of the schistosome parasite [26–28]. The provision of safe water supply is one important approach to reduce the need for contact with contaminated water bodies and diminish the risk of schistosomiasis transmission [29].

A study carried out in 2014 in Munyenge, an endemic foci located in the mount Cameroon area, revealed that *S. haematobium* infection is common among pregnant women and regular contact with stream and long duration in contaminated water sources increased risk of infection. We suggested that provision of piped water and health education will decrease disease incidence and intensity [6]. More so, a recent study by Ebai et al. [30] in some neighbouring villages to Munyenge showed that access to piped water protected individuals living in these communities from UGS. Thus, following scale up of piped water supply from 2014 to 2017 in Munyenge, this study investigated the prevalence and risk factors of UGS in pregnancy for an epidemiological update. Secondly, to assess the impact of increase piped water supply, we compared stream usage, stream contact behaviour and *S. haematobium* infection rate and intensity with the findings of 2014.

## Methods

### Study area

**Fig. 1:**
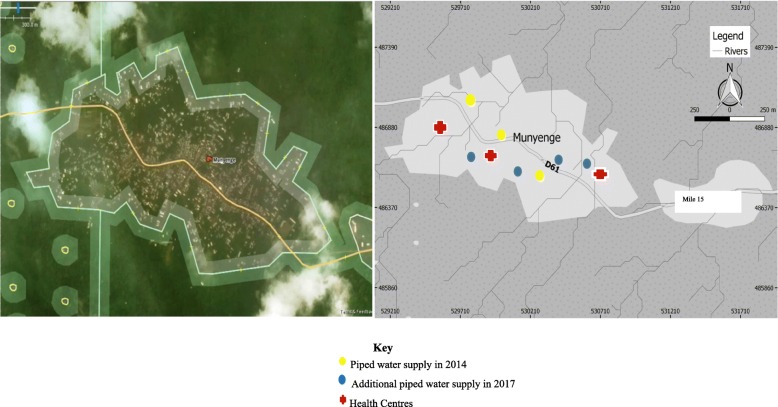
Map showing distribution of piped water sources in Munyenge between 2014 and 2017. (Source: Google Satellite Maps (ArcGlobe, ESRI®) adapted to show available health centres and communal piped water sources

**Fig. 2:**
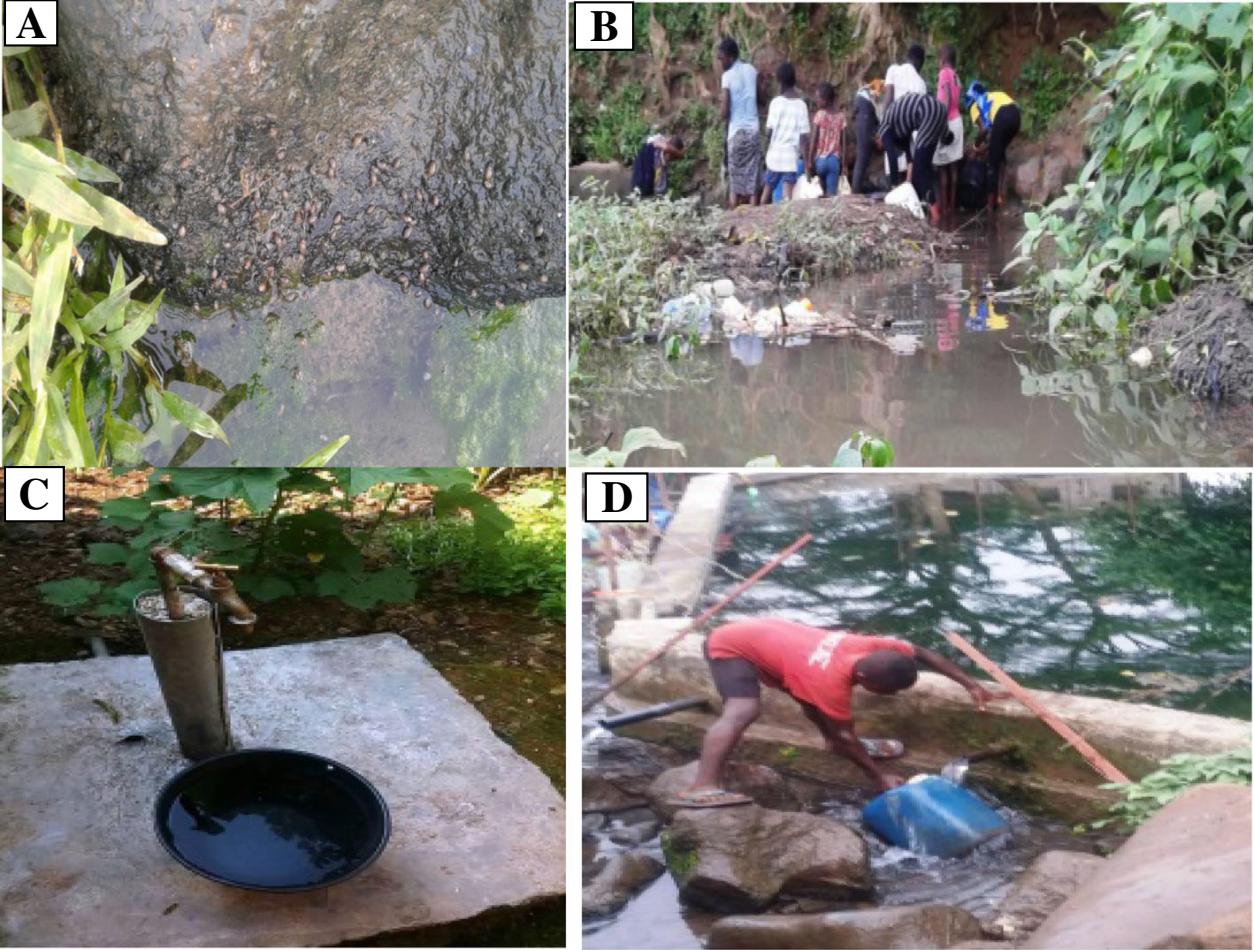
**a**: Snail infested stream, **b**: Some stream contact activities, **c** & **d**: Piped water sources

The study was carried out in Munyenge, a village located in the Bafia health area about 27 km from Muyuka town, South West Region, Cameroon. Bafia Health Area is an endemic focus for UGS, which is found in the Mount Cameroon Area. This health area is made up of three rural communities: Ikata, Bafia, and Munyenge. Munyenge has a heterogenous population of about 13,127 inhabitants (Delegation of Public Health, South West Region, 2017) with farming as the principal occupation. The characteristics of the study area has been described elsewhere [6, 31]. This community has four health centres, of which three provide antenatal care and delivery services for the local population. In Munyenge, piped water sources have increased from three to seven between 2014 and 2017 (Figs. 1 and 2). Nonetheless, access to safe water is still poor due to long distances to improved water source and water user fees that influence water use patterns and health benefits offered by improved water sources (personal observation). Consequently, the local population still makes frequent use of the streams for their daily needs.

### Study design

This was a cross sectional study that involved pregnant women, who were enrolled consecutively between November 2016 and January 2018.

### Study population

Pregnant women in their third trimester, who reported for antenatal care (ANC) at any of the three health centres (Government Integrated Health centre (HC), Banga Annex HC, Trans African HC) were enrolled in the study. Prior to enrolment, each participant provided an informed consent.

### Population sample size determination

The minimum sample size was computed using the formula by Bryan [32] based on the *S. haematobium* infection prevalence of 46.8% in pregnancy reported from an epidemiological baseline study in Munyenge by Anchang-Kimbi et al. [6].

$$ \mathrm{n}=\frac{{\mathrm{z}}^2\upalpha /2\mathrm{pq}}{{\mathrm{d}}^2} $$

n = minimum sample size required

z = 1.96 is confidence level test statistic at the desired level of significance

p = 0.468 is the proportion of UGS in pregnancy

q = 1-p = is the proportion negative for UGS

d = the acceptable error willing to be committed

$$ \mathrm{n}=\frac{(1.96)^2\times 0.468(0.532)}{(0.05)^2}=382 $$

A sample size of at least 382 was determined to be adequate to detect a 5% change in the prevalence of UGS. However, due to logistics, we had a sample size of 368 pregnant women which is well above 95% of the expected sample size calculated.

### Administration of questionnaire

The study participants were interviewed by a field researcher using a validated questionnaire to record socio-emographic information (age, residence), socio-economic indicators (marital status, educational level and occupation), gynaecologic/obstetric history (gravidity, gestational age) and questions related to schistosomiasis; household water source (stream or piped water), frequency of contact with water source, stream activities (domestic chores and bathing (measures of intensity of contact with water)). In addition, the questionnaire included questions related to knowledge about schistosomiasis etiology, transmission, clinical manifestation, prevention and control. Knowledge on UGS was scored on 4 points as described by Folefac et al. [33]. Briefly, one point was allocated for a correct response and no point for a “I don’t know” or wrong answer. A knowledge score of < 2, 2–3 and > 3 was considered as poor, average and good respectively.

### Parasitology

#### Sample collection and processing

About 20 ml of terminally voided urine sample was collected from consented participants into sterile, dry, leak-proof transparent, pre-labelled urine bottles. Women were instructed to collect urine between 10 am and 2 pm. Urine samples were immediately processed and analysed at the laboratory unit of the health facility. Haematuria was determined by visual observation of urine samples and urinalysis reagent strips (Mission® Expert-USA). *Schistosoma. haematobium* eggs were obtained and identified using the filtration technique and microscopy respectively as reported elsewhere [6]. A pregnant woman was diagnosed with UGS when she was positive by microscopic examination of urine filtrate and/or urine reagent strip. The infection intensity was classified as light (< 50 eggs/10 ml of urine) or heavy (≥50 eggs/10 ml of urine) as defined by the World Health Organization [7].

#### Data management and statistical analysis

Questionnaires were checked for the correct use of codes and completeness. Data were coded, validated and analysed using SPSS version 22.0 (SPSS, Inc., Chicago, IL, USA). The statistical tests performed included the Pearson’s chi-square for comparison of proportions. Bivariate analysis was performed to identify the factors associated with *S. haematobium* infection to be included in the multivariate logistic regression for analysis of risk factors for UGS. Variables that had a *p*- value < 0.20 in bivariate analysis or biological plausiblility were included in the multivariate logistic regression model. In order to assess the impact of increase piped water sources on infection rate and intensity, relative risk reduction was calculated using a Microsoft Excel confidence interval calculator as described by the Newcombe-Wilson method [34, 35]. A *p* *-* value < 0.05 was considered significant.

## Results

### Characteristics of the study population

A total of 368 pregnant women were enrolled into the study. The mean age of the study participants was 25 ± 5.8 years (Range: 15–42 years). The characteristics of the study participants are shown in Table 1. The majority of the women were older (> 25 years), married and had attained at least a secondary level of education. Single women were predominantly younger (≤ 25 years) (72.5%; 71/98) while, more married women were older (> 25 years) (53.3%; 144/270). The difference was statistically significant (χ^2^ = 24.17; *P* < 0.001). A greater proportion of the women were housewives. The women obtained their water from the stream and piped water sources for personal and domestic purposes. However, stream usage was predominant (76.1%). Among the 368 women, 16.2% (54) reported piped water as their only source of water, 53.3% (178) had stream as their only source of water and 30.5% (102) used both piped and stream water. No association was found between socio-demographic factors and water source type but, women who used piped water (36.4%: 102/280) were less likely (aOR = 0.34, 95% CI: 0.21–0.57, *p* < 0.001) to use the stream when compared with those who reported no access to piped water (63.6%; 178/280). Generally, women made fewer contact (< 3 times/week) with stream. Although not statistically significant (χ^2^ = 2.94; *P* = 0.086), those who reported stream as the only source of water made more contacts (≥ 3 times/weeks) (75%; 33/44) than those who used both sources (25%; 11/44). Among the women who reported stream as a source of water, a majority of them visited the stream for domestic purposes and one- third of the women equally reported bathing (Table 1). With regard to knowledge on UGS, a greater percentage of the women had poor perception of the disease symptoms and its association with water contact.

**Table 1:** Characteristics of the study participants

Variable	Characteristics	(n)	(%)
Age Group (Years)	≤20	81	22.0
	21–25	116	31.5
	> 25	171	46.5
Gravidity	Primigravidae	115	31.2
	Secundigravidae	90	24.5
	Multigravidae	163	44.3
Marital status	Single	98	26.6
	Married	270	73.4
Educational level	Primary	156	42.4
	Secondary	212	57.6
Occupation	Housewife	125	34.0
	Student	80	21.7
	Farmer	74	20.1
	Business	89	24.2
Stream usage	Yes	280	76.1
	No	88	23.9
Stream contact activity	Domestic contact and bathing	89	31.7
	Domestic contact only	191	68.2
Stream visits per week	< 3 times	236	64.1
	≥3 times	44	12.0
Type of water source	Stream only	178	53.3
	Piped and stream	102	30.5
	Piped only	54	16.2
Knowledge on UGS	Poor	246	66.8
	Average	29	7.9
	Good	93	25.3

### The prevalence and risk factors of *S. haematobium* infection

Eighty-two (22.3%; 95% CI: 15.8–22.0) of the 368 pregnant women enrolled were positive for UGS. Excretion of ova in urine was recorded for 72 (19.6%; 95% CI: 18.3–26.8) women among whom 23 (32%) had heavy (≥50 eggs/10 ml of urine) infection, while 49 (68%) had light (< 50 eggs/10 ml of urine) infection. The prevalence of microhaematuria was 14.7% (54/368) of which, 2.7% (10) of the women were positive for microhaematuria only. Using microscopic urine examination as gold standard, the specificity and sensitivity of microhaematuria in the diagnosis of *S. haematobium* infection were 96.6% (95% CI: 93.9–98.2) and 61.1% (95% CI: 49.6–71.5), respectively.

In bivariate analysis, there was an association between the prevalence of infection and marital status (*P* < 0.001), water source type (*P* < 0.001), stream activity (*P* < 0.001) and frequency of contact with stream (*P* < 0.001). All women who had piped water as the only source of water were negative for UGS (Table 2). On the other hand, no association was seen between maternal age, gravidity status, educational level, occupation and infection (Table 2). All four factors associated with UGS were retained by multiple regression model analysis (Table 2). Maternal age and educational level were included in the final model based on the biological plausibility of these factors.

**Table 2:** Risk factors associated with *S. haematobium* infection among pregnant women in Munyenge

Variable	Category	S. *haematobium* Positive %(n)	Unadjusted OR (95% CI)	^a^Adjusted OR (95% CI)	*P* value
Age Group (years)	≤20	28.4(23)	1.54(0.84–2.83)	0.88(0.37–2.09)	0.772
	21–25	20.7(24)	1.01(0.57–1.82)	0.99(0.49–2.02)	0.984
	> 25	20.5(35)	REF	REF	
	χ^2^; *P* value	2.24; 0.326			
Gravidity	Primigravidae	24.3(28)	1.37(0.77–2.44)	NA	NA
	Secundigravidae	25.6(23)	1.46(0.79–2.70)		
	Multigravidae	19.0(31)	REF		
	χ^2^; *P* value	1.84; 0.398			
Marital Status	Single	35.7(35)	3.02(1.78–5.13)	2.24(1.04–4.79)	0.038
	Married	17.4(47)	REF	REF	
	χ^2^; *P* value	13.91; < 0.001			
Educational level	Primary	25.0(39)	1.47(0.87–2.46)	2.00(1.04–3.85)	0.039
	Secondary	20.3(43)	REF	REF	
	χ^2^; *P* value	1.15; 0.283			
Occupation	Housewife	21.6(27)	1.26(0.63–2.50)	1.47(0.65–3.33)	0.360
	Student	31.2(25)	2.07(1.01–4.25)	2.50(0.86–7.30)	0.094
	Farmer	21.6(16)	1.26(0.58–2.73)	1.71(0.67–4.36)	0.260
	Business	15.7(14)	REF	REF	
	χ^2^; *P* value	5.97; 0.113			
Water source	Stream only	33.1(59)	1.70(0.97–2.95)	1.49(0.79–2.81)	0.224
	Stream and piped water	22.5(23)	REF	REF	
	Piped water only	0(0)	–	–	
	χ2; *P* value	24.89; < 0.001			
Stream contact activity	Domestic contact + Bathing	48.3(43)	3.64(2.1–6.28)	3.31(1.83–6.01)	< 0.001
	Domestic contact only	20.4(39)	REF	REF	
	χ^2^; *P* value	22.81; < 0.001			
Stream visits per week	< 3 times	24.6(58)	0.27(0.12–0.53)	0.35(0.17–0.74)	0.006
	≥3 times	54.5(24)	REF	REF	
	χ^2^; *P* value	16.08; < 0.001			

*χ*^2^ = Pearson Chi-square test; OR = odd ratio; ^a^OR = adjusted OR using multivariate regression analysis

Single status increased risk for infection by 2.2 times (95% CI: 1.04–4.79) when compared with the married status. Equally, women with a primary - level education were at higher risk (aOR = 2.0; 95% CI: 1.04–3.85) of having UGS than those with at least secondary-level education. Furthermore, higher odds (aOR = 3.3; 95% CI: 1.83–6.01) of having UGS were identified among women who carried out both domestic activity and bathing in the stream. On the other hand, less frequency of contact with water (< 3 times per week) (aOR = 0.35, 95% CI = 0.17–0.74) was associated with decreased risk of infection.

### Changes in stream usage and contact behaviour, *S. haematobium* infection rate and intensity after scale-up of piped water sources between 2014 and 2017

**Fig. 3:**
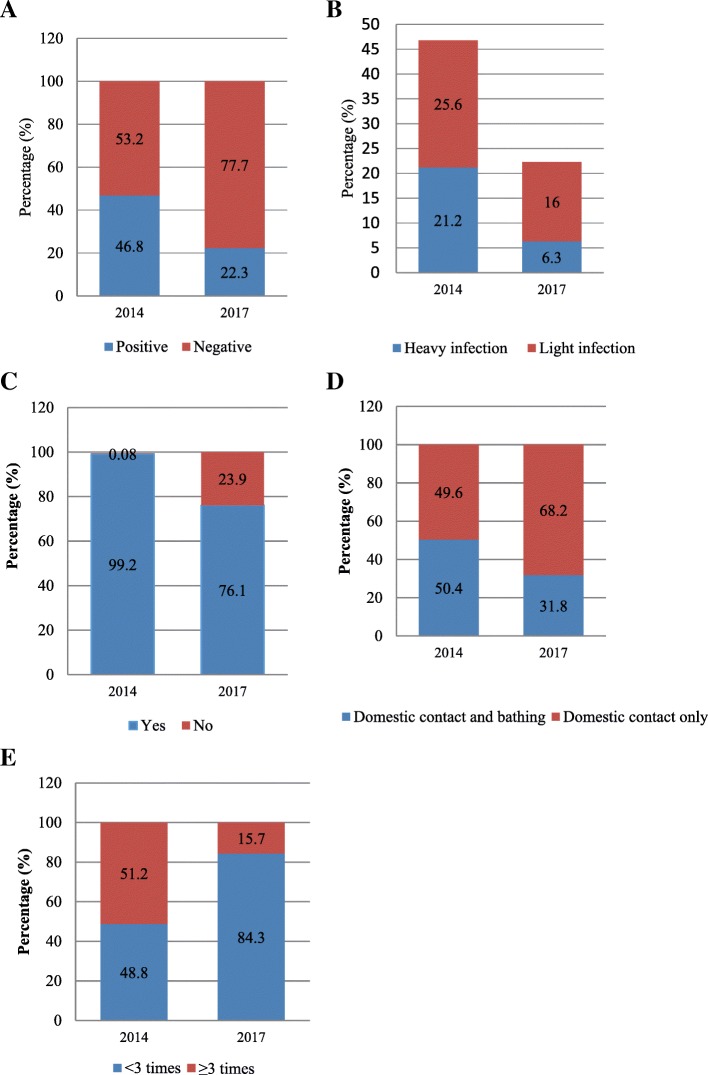
Changes in **a**: prevalence of *S. haematobium* infection **b**: intensity of infection **c**: stream usage **d**: stream contact activity **e**: stream frequency/week between two cross-sectional surveys carried out between 2014 and 2017 (Data for 2014 have been published by Anchang-Kimbi et al. [6])

About 42 % (42.4%; 156/368) of the study participants reported use of safe water after more communal piped water installations. In comparison with reports of 2014, changes in stream usage (stream contact) and frequency of contact with stream, stream activity, prevalence and intensity of UGS were observed (Fig. 3 and Additional file 1). Two hundred and eighty women (76.1%) reported stream water contact during the period of study (Table 1) while, in 2014, 99.2% of pregnant women used the stream as main source of water. Stream usage reduced by 23% (RRR = 0.23, 95% CI = 0.19–0.28). Equally, there was a decrease in the frequency of contact with water. Compared with 2014, frequent visits (≥ 3 visits/week) to the stream reduced by 69% (RRR = 0.69, 95% CI = 0.59–0.77) in 2017. Similarly, bathing activity (a measure of intense contact with water) in streams decreased by 37% (RRR = 0.37, 95% CI = 0.22–0.49). A decline in the prevalence and intensity of *S. haematobium* infection was observed among pregnant women. Infection rate decreased from 46.8% in 2014 to 22.3% in 2017 (RRR = 52, 95%, CI = 40–62%) With regards to infection intensity, cases with heavy infection decreased by 71% (RRR = 0.71, 95% CI = 0.53–0.81; *P* < 0.001) as well as the prevalence of light infection (RRR = 0.37, 95% CI = 0.14–0.54) (Additional file 1).

## Discussion

Munyenge village is an established endemic focus for UGS. [6, 30, 36]. The establishment of safe water is an essential prerequisite for schistosomiasis control in endemic areas since, the prevention of schistosmiasis is achieved by reducing contact with schistosoma-infested water [23]. The present study is a follow-up survey which reports on the prevalence and risk factors of *S. haematobium* infection among pregnant women living in Munyenge, following the scale-up of communal piped water sources between 2014 and 2017. The impact of increased piped water sources was evaluated by assessing changes in stream contact patterns, prevalence and intensity of infection.

This study carried out in Munyenge, in 2017, revealed a reduced prevalence of *S. haematobium* infection among pregnant women by 52%. In addition, we recorded a decreased in the number of cases of heavy infection. Interestingly, our study revealed that the use of piped water eliminated the risk of infection among pregnant women who completely stopped using stream water but very little effect on those who reported partial use of safe water. Similarly, the protective role of access to piped water has been reported by Ebai et al., [30] in a survey carried out in Ikata-Likoko area which, are neighbouring communities to Munyenge in the Bafia health area. Equally, studies in other endemic areas have shown that safe water supplies were associated with significantly lower odds of schistosomiasis [29, 37–42]. In Brazil, a communal water supply was shown to reduce the prevalence of the disease [37]. In Egypt, even the partial use of safe water markedly lowered the prevalence of *S. mansoni and S. haematobium* [38]. A recent study by Tanser et al. [39] reported a UGS prevalence of 16.8% following scale-up of piped water in rural South Africa which was markedly lower than the overall prevalence of 60–70% recorded in the same area thirty years earlier. Also, a cohort study carried out in the same area showed that children living in communities with a high coverage of piped water were eight times less likely to be infected relative to those living in areas with little or no access to piped water [39]. Introduction of safe water supplies into a community offers protection from schistosomiasis infection either directly or indirectly. It offers direct protection to individuals with access to safe water by reducing their contact with infested water bodies through household domestic water collection activities. Secondly, the scale-up access to safe water will confer indirect protection to members of a community in a manner that is analogous to the concept of ‘herd immunity’. Herd immunity confers protection through a reduction in the number of contacts that infected individuals have with open water bodies leading to a decrease in the overall levels of intensity of infection in the surrounding community [43].

A water supply system that is either communal or household lowers the degree of contact with infested water in a community and people change their behaviour after safe water becomes available [38, 41, 42, 44]. With increased piped water sources in Munyenge, stream usage, frequent contact with stream and prolonged duration of water contact (bathing and domestic activities in the stream) decreased significantly by 23, 69 and 37% respectively. However, in the present study, piped water usage was associated with reduced stream usage and not changes in stream contact behaviour. The change in stream contact behaviour observed may be attributed to some level of awareness on UGS in the community which, may have influenced change in attitudes of some pregnant women from bathing and washing in water bodies to stream banks or at their homes. Despite the presence of more piped water sources, a majority of the pregnant women (76%) continue to use the streams for domestic purposes. Distance from home to the taps, limited number of communal piped water sources for a dispersed population and requirement for immediate payment of piped water may account for the reliance on the stream [41, 42, 45]. It will be interesting to assess the relationship between these factors and the distribution *S. haematobium* infection in the study area.

Although the burden of UGS has declined in Munyenge over the indicated three-year period, the prevalence of infection still remains high (22.3%) among pregnant women. Coupled with the high rate of stream usage, other factors predisposed these women to the risk of *S. haematobium* infection. Marital status influenced infection outcome where single women were twice more likely to be infected with *S. haematobium* than their married counterparts irrespective of their age and gravidity status. The role of marital status in the risk of malaria parasite infection in pregnancy has also been reported [46]. Studies have shown that marriage has advantages on the health outcomes of individuals [47]. A spouse may improve economic well-being [48] as well as play an important role in monitoring and encouraging healthy behaviours [49]. Partner support may be important in limiting or preventing contact with infested water by the pregnant woman. Furthermore, infection was more common among individuals with low level of education. This is in conformity with findings of Lima E Costa et al. [44] and Bethony et al. [50] in Brazil, Khalid et al. [9] in Sudan and Salawu and Odiabo, [12] in Nigeria. Ugbomoiko et al. [51] suggested that education affects attitudes and behaviour. Individuals with low educational status are more likely to enter the stream barefoot and spend longer hours in water (exposing themselves to cercarial penetration) than their more educated counterparts. On the other hand, the self-awareness of the disease may account for the reduced prevalence level observed among women with at least a secondary level of education. Consistent with our previous findings in Munyenge, bathing and domestic activities in infested waters predisposed pregnant women to infection. Both activities increase intensity of water contact with infested water [6, 44, 52]. These findings strongly suggest that the extension of more piped water sources in this endemic area will reduce the incidence of infection by reducing the need for intense or frequent contact with infested water. However, a productive and sustainable intervention cannot be achieved without adequate education [52, 53]. We suggest that educating women during antenatal clinic visits on the harmful effects of UGS and the local risk factors of infection will help reduce frequency of water contact and thus infection risk in this endemic areas.

This study had a few limitations. This study examined the short-term effect of piped water supply on water contact patterns and transmission of *S. haematobium* infection in Munyenge. Studies have demonstrated that over long-term period, water supply facilities had little impact on the overall prevalence and intensity of infection [39, 42]. Secondly, piped water usage and stream contact behaviour were based on self- reports and not by direct observation. Analysis of spatial pattern of infection, observations of human contact at the stream and a questionnaire on water use gives a better assessment of the impact of piped supply on human water contact [39, 41, 42].

## Conclusion

Following the scale-up of communal pipe water sources in Munyenge from 2014 to 2017, the prevalence of UGS among pregnant women has decreased significantly by 52%. Equally, a reduction in stream usage, frequency and intensity of contact with stream was observed. The use of piped water reduced stream usage by pregnant women and eliminated the risk of UGS among those who completely avoided the stream. Single status, low level of education and activities that the prolong duration in water predisposed the women to *S. haematobium* infection. Despite the increase safe water sources, the majority of these women still depend on the natural source of water for their daily activities. It is obvious that expansion of piped water sources is important in interrupting stream contact but this intervention is productive and sustainable, if health education activities directed towards avoiding water contacts are achieved.

## Additional file

Additional file 1: Relative risk reduction in stream usage and contact behaviour, *S. haematobium infection rate* and intensity among pregnant women following scale-up of communal piped water sources from 2014 to 2017 in Munyenge. This file shows how much the risk of *S. haematobium* infection/intensity, stream usage and contact behaviour has reduced among pregnant women following scale-up of communal piped water sources from 2014 to 2017 in Munyenge. (DOCX 16 kb)

## Abbreviations

ANC: Antenatal care clinic
HC: Health Centre
HIV: Human Immunodeficiency Virus
RRR: Relative risk reduction
UGS: Urogenital schistosomiasis
