# Supplementary material for: Schistosoma haematobium infection status and its associated risk factors among pregnant women in Munyenge, South West Region, Cameroon following scale-up of communal piped water sources from 2014 to 2017: a cross-sectional study
Source: BMC Public Health. 2019 Apr 11;19:392. doi: 10.1186/s12889-019-6659-7 (PMC6458650; doi:10.1186/s12889-019-6659-7)
Supplement: Supplementary file 1 — Relative risk reduction in stream usage and contact behaviour, S. haematobium infection rate and intensity among pregnant women following scale-up of communal piped water sources from 2014 to 2017 in Munyenge. This file shows how much the risk of S. haematobium infection/intensity, stream usage and contact behaviour has reduced among pregnant women following scale-up of communal piped water sources from 2014 to 2017 in Munyenge. (DOCX 16 kb) [file 12889_2019_6659_MOESM1_ESM.docx]

**Additional file 1:** **Relative risk reduction in stream usage and contact behaviour, *S. haematobium* infection rate and intensity among pregnant women following scale-up of communal piped water sources from 2014 to 2017 in Munyenge**

| **Variable** | **Category** | **2014***  **(N = 250)**  **n (%)**  **95% CI** | **2017**  **(N = 368)**  **n (%)**  **95% CI** | **RR**  **95% CI** | **RRR 95% CI** | **ARR**  **95% CI** |
| --- | --- | --- | --- | --- | --- | --- |
| **Stream usage** | Yes | 248 (99.2)  ( 97.1 – 99.8) | 280 (76.1)  (71.5 – 80.2) | 0.77  (0.72 – 0.81) | 0.23  (0.19 – 0.28) | 0.23  (0.19 – 0.28) |
|  | No | 2 (0.8)  (0.0 -3.0) | 88 (23.9)  (19.8 – 28.5) | 29.9  (7.4 – 120 .3) | NA | NA |
| **Stream frequency per week** | <3 visits | 122 (48.8)  ( 42.6 – 55) | 236 (84.3)  (79.6 – 88.1) | 1.72  (1.51 – 1.98) | NA | NA |
|  | ≥3 visits | 128(51.2)  (45.0 -57.3) | 44 (15.7)  (11.9 – 20.44) | 0.31  (0.23 – 0.41) | 0.69  ( 0.59 – 0.77) | 0.36  0.28 – 0.43 |
| **Stream contact**  **activity** | Domestic activity + bathing | 126 (50.4)  (44.2 -56.5) | 89 (31.8)  (26.6 – 37.5) | 0.63  (0.51 – 0.78) | 0.37  (0.22 – 0.49) | 0.19  (0.10 – 0.27) |
|  | Domestic activity only | 124 (49.6)  ( 43.5 – 55.8) | 191 (68.2)  (62.6 – 73.4 ) | 1.38  (1.19 – 1.60) | NA | NA |
| ***Schistosoma haematobium* infection status** | Positive | 117(46.8)  (40.7 – 53 ) | 82(22.3)  (18.3 – 26.8) | 0.48  (0.38 – 0.6) | 0.52  (0.4 – 0.62) | 0.25  (0.17 – 0.32) |
|  | Negative | 133 (53.2)  (47.0 – 59.3) | 286 (77.7)  (73.2 – 81.7) | 1.46  (1.29 – 1.66) | NA | NA |
| **Intensity of infection** | Heavy infection | 53(21.2)  ( 16.6 – 26.7) | 23(6.3)  (4.2 – 9.2) | 0.29   - 1. – 0.47) | 0.71  (0.53 – 0.81) | 0.15  (0.1 – 0.21) |
|  | Light infection | 64 (25 .6)  (20.5 – 31.4) | 59 (16.0)  (12.6 – 20.1) | 0.63  (0.46 – 0.86) | 0.37  (0.14 – 0.54) | 0.1  (0.03 – 0.16) |

* Data published by Anchang-Kimbi *et al*., 2017

RR = Relative risk, RRR = Relative risk reduction, ARR= Absolute risk reduction, 95%CI = 95% Confidence interval, NA = Not applicable.
